# The Role of MRI-Guided Radiation Therapy for Palliation of Mobile Abdominal Cancers: A Report of Two Cases

**DOI:** 10.1016/j.adro.2021.100662

**Published:** 2021-02-03

**Authors:** Olga L. Green, Lauren E. Henke, Alex Price, Areti Marko, Erin J. Wittland, Soumon Rudra, Hyun Kim, Sasa Mutic, Jeff Michalski, Clifford G. Robinson

**Affiliations:** Department of Radiation Oncology, Washington University School of Medicine, St. Louis, Missouri

## Introduction

Magnetic-resonance image guided-radiation therapy (MRgRT) is increasingly used in the curative setting for advanced treatment techniques like online-adaptive radiation therapy.^1^, ^2^, ^3^, ^4^, ^5^, ^6^ In contrast, for most palliative treatments, weekly imaging with x-ray–based systems (kV or MV) is considered adequate. However, for lesions involving the bowel or omentum where inter- and intrafraction motion may be pronounced, bony anatomic alignment may be insufficient, even with generous margins. The superior soft tissue imaging and flexibility of MRgRT for online adaptive radiation therapy planning may prove beneficial for such palliative cases involving mobile gastrointestinal anatomy. Here we present 2 palliative cases — a bleeding jejunal tumor and an oligometastatic omental lesion — where MRgRT was required to manage inter- and/or intrafraction motion for effective palliative treatment.

## Case 1: Presentation

A 68-year-old man with metastatic non-small cell lung cancer presented with several weeks of gastrointestinal hemorrhage requiring transfusion. Computed tomography (CT) of the abdomen revealed a distal jejunal mass centered in the upper pelvis. Upper endoscopy and biopsy confirmed metastatic adenocarcinoma consistent with lung primary. Due to comorbidities, he was ineligible for surgery and was referred to radiation oncology for urgent palliative treatment. A course of 25 Gy in 5 fractions was prescribed. Given the known substantial mobility of the small bowel, a 0.35-T MRgRT system with daily volumetric MR-guided setup and real-time cine imaging and gating during delivery was employed.^7^^,^^8^

### Case 1: Treatment planning and delivery

Because this case was urgent, simulation and first fraction of treatment occurred on the same day, 4 hours apart. The suitability of the patient to undergo MRI simulation and treatment was evaluated at time of consult (the day before simulation and treatment), via review of medical history and completion of an MRI questionnaire by the MRgRT team at our clinic.

**Figure 1:**
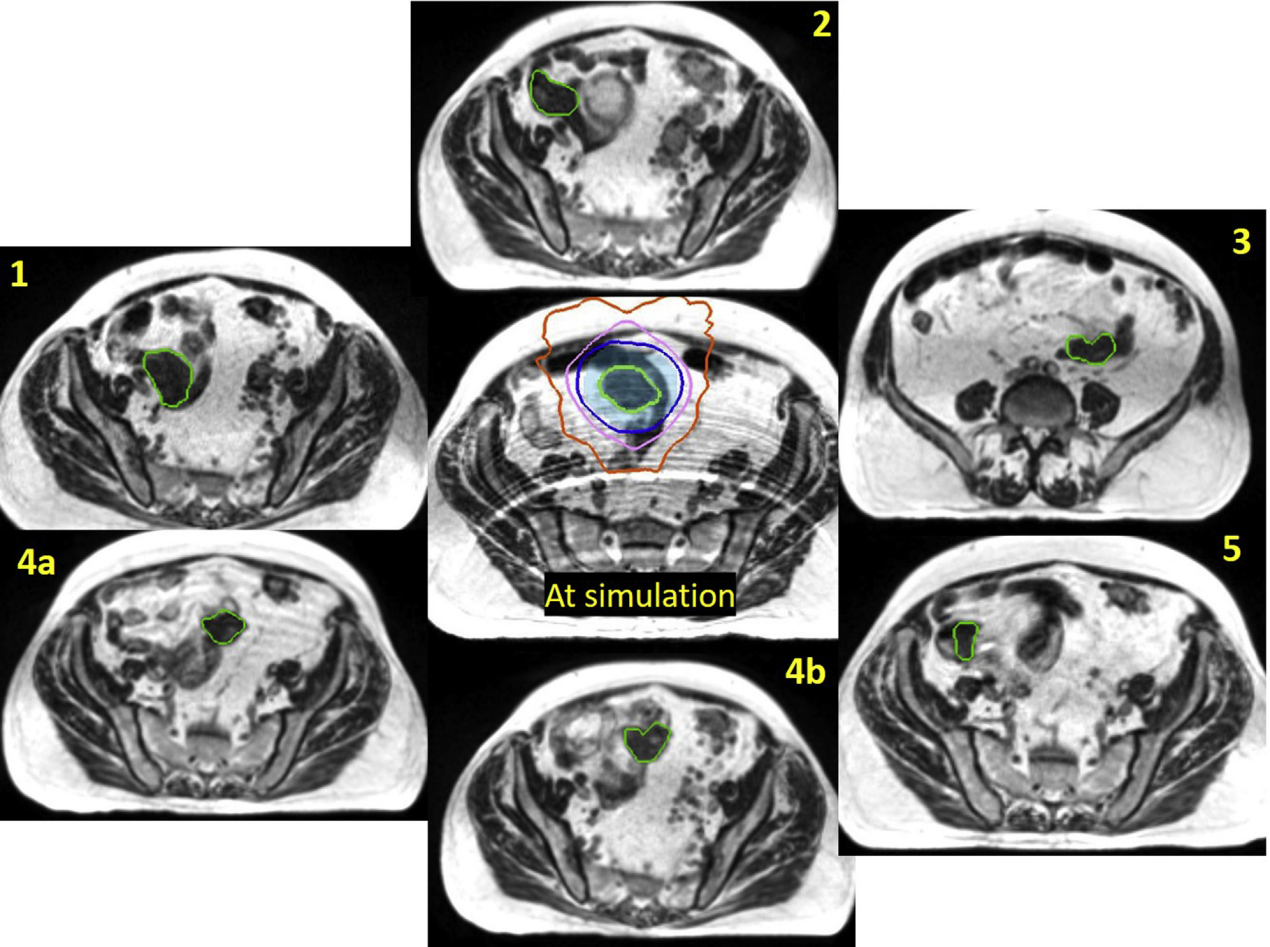
Location of the bleeding jejunal mass (green outline) as observed at simulation and each treatment fraction as noted. The simulation image also shows the 1-cm planning target volume (PTV) (light blue colorwash) and isodose lines for 100% (dark blue), 80% (pink), and 50% (orange) of the 25-Gy prescription. Real-time imaging during fraction 4 indicated that the mass had shifted, necessitating patient realignment and thus 2 images (4a and 4b). (A color version of this figure is available at https://doi.org/10.1016/j.adro.2021.100662.)

The MRgRT hybrid linear accelerator system used in this case, including characteristics of the onboard imaging unit and integrated Monte-Carlo-based treatment planned system, has been described.^8^^,^^9^ The patient was simulated supine, with arms at sides, without additional immobilization. We specifically elected against custom immobilization in this patient, given the potentially large range of motion of the tumor. Our clinic’s typical custom immobilization includes rigid fixation of the bottom MRI receiver coil to the custom mold. Had we used this typical setup, we would have been unable to move the coils, if needed, to the best imaging position, which is with the center of the coils as close to the target region of interest as possible. The MRgRT system used in our clinic allows for 3° of freedom of couch motion, thus minimizing any issue of adaptation due to gross external patient anatomy/positioning. A 3-minute, free-breathing, volumetric MRI was acquired and used as the primary planning image. The jejunal lesion was identified and contoured as the gross tumor volume (GTV). It was noted that a shorter imaging sequence would be preferred for treatment days, as although the tumor did not move with respiration during simulation imaging, there was a general abdominal wall respiratory motion artifact on the planning MRI that affected visual evaluation of the bowel and ease of target identification (Fig 1 center). The planning target volume (PTV) was defined as a 1 cm isotropic GTV expansion. A static conformal plan was created, taking advantage of the doubly focused multi-leaf collimators (MLCs) of the MRgRT system to ensure a compact dose distribution. The optimized 3-dimensional conformal treatment plan used 12 gantry angles, with 1 segment per angle. The location of the lesion at the time of simulation and plan isodose distributions are shown in Figure 1.

For patient setup at fraction 1, a 17-second single end-exhale breath-hold volumetric scan was acquired. The jejunal lesion was noted to have moved substantially from time of simulation, due to peristalsis. An adjustment of the patient’s position via couch translations necessitated a re-evaluation of the dose distribution after adjusting the assigned relative electron density to the patient’s habitus. The dose distribution from the original plan was then recalculated onto the new MRI data set; the PTV was inadequately covered by the original plan. The plan was therefore adjusted using beam weight optimization. This process left intact the beam gantry angles and MLC positions but changed the monitor units to produce a dose distribution similar to the original plan, taking into account the now-different path-lengths of the beams. A secondary dose calculation was performed as part of pretreatment quality assurance for the new plan. Online replanning was rapidly performed while the patient remained on the table in the treatment position. Similar plan adjustments were required at fractions 3 and 5. The criteria for adaptation was occurrence of gross tumor motion beyond the 1 cm PTV expansion and where shift-to-match location was either not possible due to patient collision with the MRI bore (due to large shift magnitude) or because it resulted in large incongruencies between electron density maps such that the predicted dose, when accounting for these changes, was less than 90% of prescription to 95% of the PTV. A single iteration of reoptimization was required for each adaptive plan, which is typical of our clinic’s MRgRT system for both intensity modulated radiation therapy and optimized conformal plans. Before beam-on, a sagittal plane was selected for real-time target monitoring and automatic beam-gating directly upon the target volume using a deformation registration algorithm that has been previously described.^7^ The time from image acquisition to completion of treatment delivery was about 20 minutes for each fraction, including the time for evaluation for and need for adaptation. Plan adaptation time was minimized in this case (~5 minutes per fraction), as the palliative dose level used (25 Gy in 5 fractions) was below our institutional 5-fraction abdominal constraint (36 Gy to 0.5 cc), obviating the need to recontour adjacent loops of bowel at each fraction.

This imaging and evaluation process was repeated for all fractions. All daily volumetric images were acquired at end-exhale breath-hold position, in a single 17-second breath-hold, minimizing any contribution of respiratory phase variability to observed interfraction motion. Additionally, all gated delivery (upon the GTV itself) was at the exhale position. After a “radiographic bowel run” to localize the tumor, setup position adjustments were needed at each fraction due to shifting locations of the jejunal tumor. During fraction 4, an additional intrafraction position adjustment was required. For the first 2 minutes of the 5-minute treatment, the lesion was well-visualized in the sagittal plane. However, during real-time cine monitoring, the appearance of the lesion abruptly changed and treatment was immediately paused. Repeat 17-second volumetric scan revealed additional peristaltic motion of the target, and a second position adjustment was performed for successful fraction completion. After completion of treatment, the patient reported resolution of melena, and his hemoglobin improved without subsequent transfusion requirement. He had no evidence of acute toxicity at 3 months follow-up.

### Case 1: Quantitative analysis

A quantitative analysis of the extent of motion of the jejunal lesion as observed on the 0.35-T MR images was performed by determining the lesion’s position relative to a stable anatomic landmark, the L5/S1 interspace. The distance from the center of this landmark to the centroid of the GTV as observed at the time of simulation was designated as the baseline. For each subsequent fraction, the GTV was contoured where observed on that day, and the centroid distance to the landmark was calculated in all cardinal directions. Comparing these distances to the baseline gave the actual distance of each instance of the location of the GTV relative to the original position at time of simulation. The results are summarized in Table 1. The minimum distance between the GTV centroid positions was 2.4 cm, greatly exceeding the 1-cm PTV expansion. The maximum distance was over 8 cm.

**Table 1:** Displacement of the centroid of the bleeding jejunal mass from its location at simulation as observed at each treatment session (fraction)∗

Position of centroid of GTV relative to simulation (cm)				
Fraction	Lateral	Longitudinal	Vertical	Magnitude
1	2.09	1.82	4.15	4.99
2	4.77	1.71	1.24	5.22
3	-5.60	-5.42	2.92	8.32
4a	-2.10	0.64	1.82	2.85
4b	-1.59	1.78	1.22	2.68
5	5.27	-0.19	1.83	5.58

*Abbreviation:* GTV = gross tumor volume.

∗ Please see text for detailed explanation.

In addition, without cine gating, the intrafraction motion of the tumor in fraction 4 would have been missed. Even with accurate initial setup, the lesion moved by 1.4 cm during delivery and would have been partially missed by the 2.5 Gy of the latter half of the fraction, resulting in a 10% dosimetric error in the unirradiated region (approximately 2.6 cm of the 4-cm lesion).

Lastly, the availability of weight optimization for a simple conformal plan allowed for restoration of acceptable dose distributions for each fraction (first, third, fifth) at which the lesion changed position enough to warrant it. It should be noted that the position of the mass at the time of the diagnostic CT was within 1 cm of the position observed at the time of simulation. This would have given a false sense of positional stability and resulted in a geographic miss if the patient had received standard, x-ray-based localization and treatment.

### Case 2: Presentation

The second case is of a 36-year-old man with oligometastatic clear cell renal cancer, originally treated with nephrectomy and stereotactic body radiation therapy to lesions of the lumbar spine (L3), sternum, and the left anterior rib. Two years after his original diagnosis, surveillance CT imaging showed a new solitary omental metastasis. Surgical resection would have required ex-lap with bowel run for intraoperative identification and removal. He was instead consented for stereotactic body radiation therapy for oligometastasis ablation, using the MRgRT system. However, when the patient came in for his simulation, the nodule could no longer be identified on either the simulation CT scan or the MRI scan performed shortly afterward. The treating physician discussed the findings with the patient and at first adopted a watchful waiting approach.

Two months after the initial simulation attempt, the patient had repeat diagnostic CT imaging for follow-up, which reidentified the growing omental lesion. The location of the lesion was markedly different between diagnostic CTs, and it was determined that the lesion had been difficult to identify on the original simulation because its position was unpredictable, from mobility of the omental curtain. The patient underwent repeat simulation that afternoon. The tumor location on the diagnostic CT scan of that morning was quite different from its location on the simulation CT in the afternoon. Therefore, the MRI simulation for MRgRT involved acquiring multiple scans at an exhale breath-hold (to eliminate breathing artifact), while moving the field of view from the upper midabdomen to the pelvis. The nodule was finally identified this way, and it was also observed that the nodule’s movement subsided as a function of time the patient was resting on the treatment couch, over the course of about 15 minutes, as the patient/bowel settled into position.

### Case 2: Treatment planning and delivery

A treatment plan was created in the MRgRT system’s treatment planning module to deliver 35 Gy in 5 fractions, with dose escalation permitted up to 50 Gy to 95% of the PTV, provided that strict abdominopelvic organ-at-risk constraints could be met. The PTV was defined as the GTV plus 1 cm isotropic expansion. The GTV was spherical, 1.6 cm in diameter, with a volume of 2.2 cc. Potential organs-at-risk primarily included the stomach, duodenum, small, and large bowel. The plan was made to be robust for daily online adaptation.

The first fraction attempt at treatment was unsuccessful—despite waiting 15 minutes for bowel settling, the nodule was not visible. It was suspected that it was too near a loop of bowel to be distinguished from the bowel itself. The patient was therefore not treated, and a second attempt at fraction 1 was made the following day. This time, after multiple scans, the lesion was located but was substantially higher in the abdomen than it had been at the time of simulation. After online replanning to account for this motion and ensure dosimetric constraints were met, treatment was delivered successfully. The nodule was monitored with real-time imaging in the sagittal plane. Fractions 2 to 5 were delivered in the first on-table attempts, with similar requirement for a radiographic bowel run to identify the lesion location each day.

One year after treatment completion, the patient has continued to be seen in follow-up clinic, with no further growth of the omental lesion. Restaging imaging has shown central necrosis of the omental lesion consistent with treatment response. No acute or late abdominopelvic toxicities have been observed, and he has not required any further systemic therapies at this time.

### Case 2: Quantitative analysis

**Figure 2:**
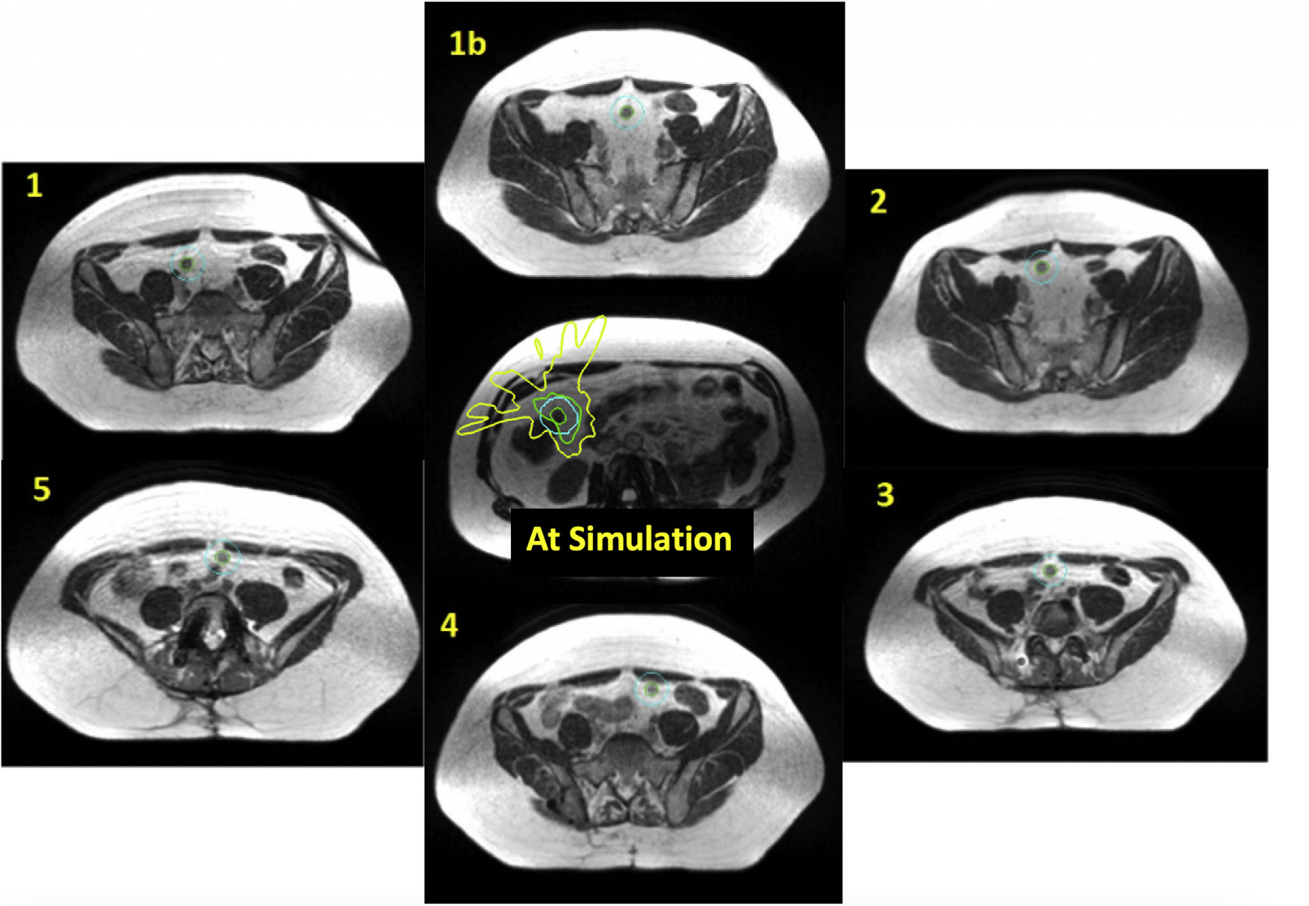
As noted in the text, the first attempts at both simulation and treatment failed due to inability to locate the nodule (green outline). At the first fraction, the nodule moved after the adaptive plan was created, and therefore another image was acquired (1b). The 1-cm planning target volume (PTV) (light blue), although adequate for stereotactic body radiation therapy (SBRT), was not enough to encompass the motion of the nodule for each fraction. (A color version of this figure is available at https://doi.org/10.1016/j.adro.2021.100662.)

The nodule was observed to move at least 2 cm each day (3 cm average) and had a maximum lateral movement of 5 cm at fraction 4. The L5/S1 interspace was chosen as in case 1 to compare the relative positions of the nodule between simulation and treatment. Results are summarized in Table 2, and the simulation image and plan as well as daily treatment images are shown in Figure 2. As with the previous case, it would be nearly impossible to treat this patient without the use of MRgRT and daily adaptation.

**Table 2:** Displacement of the 1.2-cm nodule relative to simulation at each of the 5 scheduled fractions∗

Position of centroid of GTV relative to simulation (cm)				
Fraction	Lateral	Longitudinal	Vertical	Magnitude
1	–1.97	–1.95	–1.21	3.02
2	–0.63	2.10	–0.58	2.27
3	–2.07	–2.10	–0.81	3.06
4	–4.99	0.00	–1.90	5.34
5	–1.87	1.50	–0.44	2.44

*Abbreviation:* GTV = gross tumor volume.

∗ Please see text for detailed explanation.

## Conclusions

This is the first described clinical use of MRgRT and adaptive radiation therapy for the palliative treatment of extremely mobile gastrointestinal tumors, including a hemorrhagic intestinal metastasis in an emergency setting and a solitary omental oligometastasis treated definitively. The observed motion of both tumors was beyond the boundary of commonly used PTVs, and classical palliative treatment modalities without MRgRT or the ability to perform online adaptation when needed would have resulted in partial or complete geographic treatment misses in each case. Although palliative therapy should be approached pragmatically, advanced image guidance and radiation therapy techniques like on-table adaptation may be required in some cases to achieve successful radiation therapy delivery.
